# Cesarean sections and early-term births according to Robson classification: a population-based study with more than 17 million births in Brazil

**DOI:** 10.1186/s12884-023-05807-y

**Published:** 2023-08-03

**Authors:** Aline S. Rocha, Enny S. Paixao, Flavia Jôse O. Alves, Ila R. Falcão, Natanael J. Silva, Camila S. S. Teixeira, Naiá Ortelan, Rosemeire L. Fiaccone, Laura C. Rodrigues, Maria Yury Ichihara, Mauricio L. Barreto, Marcia F. de Almeida, Rita de Cássia Ribeiro-Silva

**Affiliations:** 1 School of Nutrition, Federal University of Bahia (UFBA), Araújo Pinho - No. 32, Canela, Salvador, Bahia Brazil; 2 Center for Data and Knowledge Integration for Health (CIDACS), Oswaldo Cruz Foundation, Salvador, Brazil; 3 Faculty of Epidemiology and Population Health, London School of Hygiene and Tropical Medicine, London, UK; 4 Institute of Collective Health, Federal University of Bahia (ISC/UFBA), Salvador, Brazil; 5 Barcelona Institute for Global Health, Hospital Clínic, Barcelona, Spain; 6 Department of Statistics, Federal University of Bahia (UFBA), Salvador, Brazil; 7 School of Public Health, University of São Paulo (USP), São Paulo, Brazil

**Keywords:** Early-term births, Cesarean section, Robson classification

## Abstract

**Background:**

Cesarean section (CS) rates are increasing worldwide and are associated with negative maternal and child health outcomes when performed without medical indication. However, there is still limited knowledge about the association between high CS rates and early-term births. This study explored the association between CSs and early-term births according to the Robson classification.

**Methods:**

A population-based, cross-sectional study was performed with routine registration data of live births in Brazil between 2012 and 2019. We used the Robson classification system to compare groups with expected high and low CS rates. We used propensity scores to compare CSs to vaginal deliveries (1:1) and estimated associations with early-term births using logistic regression.

**Results:**

A total of 17,081,685 live births were included. Births via CS had higher odds of early-term birth (OR 1.32; 95% CI 1.32–1.32) compared to vaginal deliveries. Births by CS to women in Group 2 (OR 1.50; 95% CI 1.49–1.51) and 4 (OR 1.57; 95% CI 1.56–1.58) showed the highest odds of early-term birth, compared to vaginal deliveries. Increased odds of an early-term birth were also observed among births by CS to women in Group 3 (OR 1.30, 95% CI 1.29–1.31), compared to vaginal deliveries. In addition, live births by CS to women with a previous CS (Group 5 - OR 1.36, 95% CI 1.35–1.37), a single breech pregnancy (Group 6 - OR 1.16; 95% CI 1.11–1.21, and Group 7 - OR 1.19; 95% CI 1.16–1.23), and multiple pregnancies (Group 8 - OR 1.46; 95% CI 1.40–1.52) had high odds of an early-term birth, compared to live births by vaginal delivery.

**Conclusions:**

CSs were associated with increased odds of early-term births. The highest odds of early-term birth were observed among those births by CS in Robson Groups 2 and 4.

**Supplementary Information:**

The online version contains supplementary material available at 10.1186/s12884-023-05807-y.

## Background

Cesarean section (CS) rates have been steadily increasing in recent decades, particularly in low- and middle-income countries (LMICs) [1]. Brazil is one of the countries with the highest CSs rates in the world (56%) [1], and almost 90% of these are among women who receive private healthcare during childbirth [2].

Brazil has a delivery care model characterized by excessive use of obstetric and neonatal interventions. However, from the 1980s onwards, a series of government policies and programs were instituted to change this situation [3]. In this context, programs were created to improve the quality of care during labor and birth, such as the Rede Cegonha strategy, in the public sector. This program promotes the implementation of a new care model for labor and birth [4]. The Parto Adequado project, in the private sector, has the main objective of reducing cesarean sections in private services in Brazil [5]. Despite past and current initiatives, the growing trend of caesarean sections remains.

CSs may be associated with lower maternal and perinatal mortality and morbidity when performed for medical reasons [6]. On the other hand, a cesarean delivery without a medical indication (for example elective and repeat CS) may lead to negative health outcomes [7], such as early-term births (37–38 weeks of gestation) [8–10]. Early-term live births have higher neonatal morbidity, admission to Neonatal Intensive Care Units (NICU), respiratory complications at birth, neonatal mortality, and delays in long-term developmental outcomes compared to 39–41 weeks of gestation [11–15]. In addition, early-term births may produce several economic consequences related to the cost of health, social, and educational services [16].

Early-term birth rates are high worldwide, ranging from 15.6 to 30.8% in high-income countries [17]. However, there is a lack of data from LMICs [8]. In Brazil, early-term births represent 35% of all live births [11]. A study showed that the prevalence of early-term births was 1.64 higher in municipalities with ≥ 80% CS rates, compared to those with < 30% [8]. However, this study was not able to evaluate CS indications.

The Robson classification system is a useful standard to monitor and compare CS rates globally [18]. A low level of CS clinical need and rates is expected in Groups 1 to 4 (women at term, cephalic presentation, and a single fetus). On the other hand, a higher level of CS need and rates is expected in Groups 5 (women with a previous CS) and 6 to 10 (women with twins, breech position, other abnormal presentation, or preterm births) [19].

Understanding the relationship between CSs and early-term births in a country with one of the highest CS rates in the world has the potential to inform new strategies to optimize CSs use and reduce early-term births. Thus, this study evaluated the association between CSs and early term births in different Robson groups using data from more than 17 million live births in Brazil.

## Methods

### Study design and population

This population-based cross-sectional study used routine registration data from the Brazilian live birth information system (Sistema de Informações sobre Nascidos Vivos, SINASC), between January 1, 2012 and December 31, 2019.

SINASC includes information on mothers and newborns throughout Brazil, including the mother’s name, place of residence, age, marital status, education, obstetric history (previous CS or vaginal deliveries), prenatal care, pregnancy characteristics (length of gestation, type of delivery, and fetal presentation) and newborn characteristics (singleton, multiples, birth weight; presence of congenital anomalies, and gestational age). The SINASC form does not record the number of previous births and, therefore, so we used the number of previous pregnancies as a proxy for parity.

Birth certificates are the instrument which feeds SINASC. This must be completed throughout the national territory for all live births [20]. The birth certificates, processed by the notifying units, are sent and consolidated by the national birth database [21]. SINASC is considered of adequate quality, acceptable, representative, opportune, stable, and capable of meeting the intended objective: to subsidize maternal and child care planning [22].

At term live births (37 to 41 completed weeks of gestation) for women aged 14 to 49 were included in the study. Live births weighing < 500 g and birth anomalies (potentially related to the CS indication) were excluded. We also excluded records without detailed information on variables used in the Robson classification: type of delivery, previous pregnancy, gestational weeks at delivery, number of fetuses, delivery onset (prepartum CS, induced, or spontaneous vaginal delivery), and history of previous CSs.

### Outcomes

The primary outcome in this study was early-term (37 and 38 weeks gestation) compared to full and late-term births (39 to 41 weeks of gestation). The main exposure variable was a CS compared with vaginal delivery. In order to study the association between a CS and early-term birth, we used the Robson classification system to compare groups of live births from women with expected high and low CS rates. Six obstetric characteristics: previous pregnancy, gestational weeks at delivery, number of fetuses, delivery onset (pre-labor CS, induced or spontaneous vaginal delivery), previous CS and fetal presentation were used to create 10 mutually exclusive Robson groups. Group 10 was not included in the study since it included preterm births (< 37 weeks of gestation).

### Statistical analysis

Socioeconomic, maternal, and birth characteristics were summarized using frequency distributions. Logistic regression was used to estimate the odds ratio (OR) and 95% confidence intervals (95% CI) for the association between cesarean section and early-term birth.

We used propensity score matching (PSM) to control for confounding. Matching was based on risk factors, including race/ethnicity (white, black, Asian, mixed-race, and indigenous), maternal education (none, 1–3, 4–7, 8–12, and > 12 years of education), marital status (married/civil partnership, single, widowed, or divorced), number of prenatal appointments (none, 1–3, 4–6, and ≥ 7 consultations), maternal age at delivery (14–19; 20–34; and 35–49 years old), newborns’ gender (male or female) and year of birth (2012–2019). The propensity score was obtained via multiple logistic regression and matched using the nearest neighbor algorithm (1:1) without replacement and at a 0.1 caliper [23]. The analyses were conducted separately for each Robson group and the study population as a whole. Since a vaginal birth was more common than a CS in Robson Groups 1, 3 and 4, we generated matched pairs by selecting a vaginal birth for each CS. Conversely, for Groups 2, and 5 to 9, where a CS was more common, we selected a CS for each vaginal birth. In the analysis for the entire study population, we selected a vaginal delivery for each CS. We estimated the population attributable fraction (PAF) using the punaf package in Stata, which uses a logistics regression method and provides PAF and 95% CI [24]. For the PAF calculation, we used the OR obtained after the matching.

In addition, to assess differences between the early-term birth categories, we performed additional analyses, separately considering live births at 37 and 38 weeks of gestation (supplementary material). Considering the different distributions of CS rates across the geographic areas of the country, we carried out complementary analyses for the North, Northeast, Southeast, South and Central-west geographic regions. These additional multivariate logistic regression models were conducted according to the same method as the main analyses.

In order to test the robustness of our results, we performed additional analyses using a finer caliper (0.05) for matching, and crude and adjusted logistic regression for the same confounders used in the propensity score.

All analyses were performed using STATA version 15.0 (Stata Corporation, 153 College Station, USA).

## Results

**Fig. 1:**
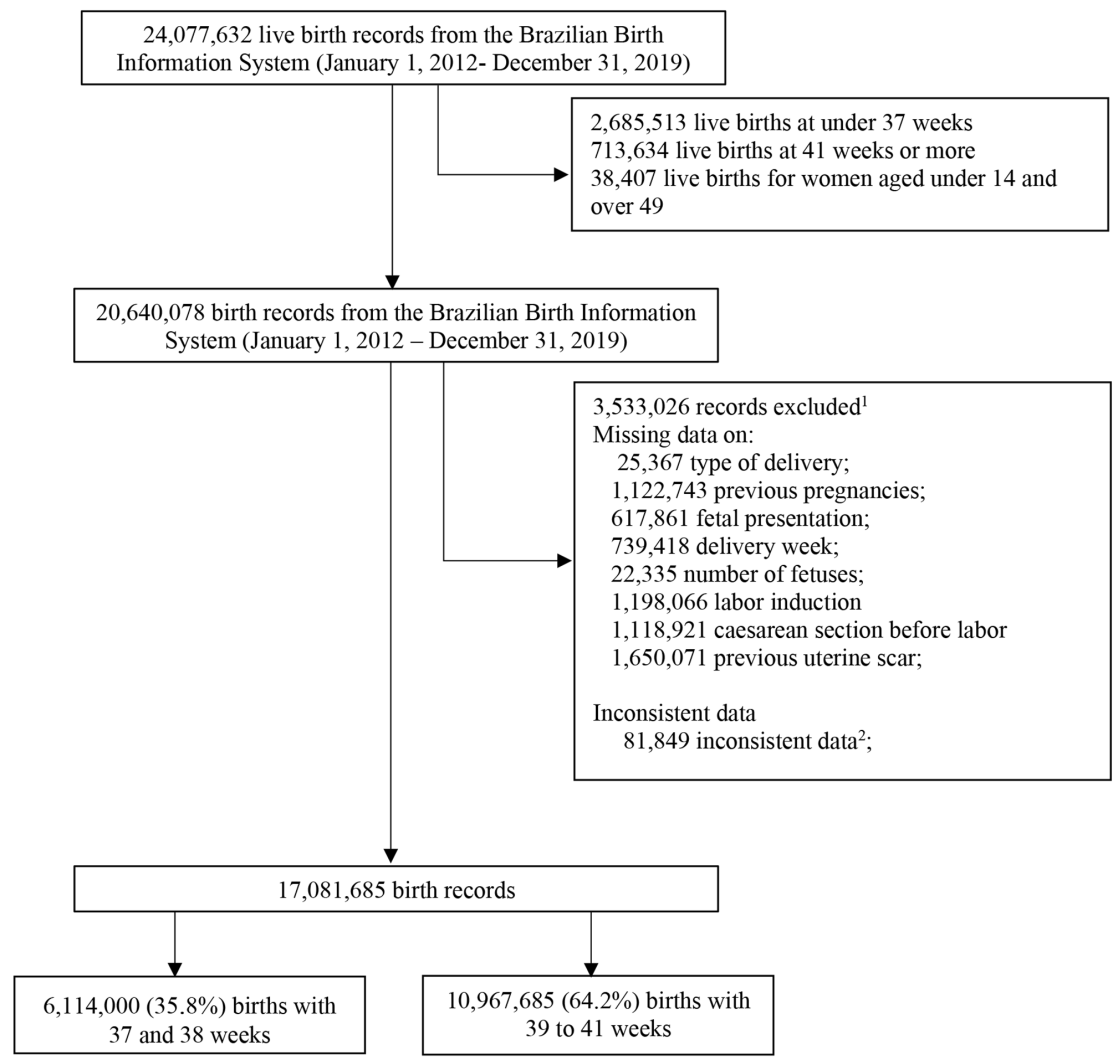
Study population flow diagram. ^1^The reasons for exclusion do not form a total of 3,558,215 since a record may lack multiple variables. ^2^Contradictory data, such as records with no previous pregnancies and previous vaginal and caesarean deliveries

During the study period, 24,077,632 live births were registered on SINASC. Of these, 17,081,685 (70.94%) were included in this study. Early-term births accounted for 6,114,000 (35.79%) of all the live births included in the study (Fig. 1). Compared to those with 39–41 weeks of gestation, early-term births were more frequent among mothers aged ≥ 35 years, married/in a civil partnership, with a higher level of education (12 years and over), were white, had a CS delivery, lived in wealthier geographic regions (Southeast, South and the Central-West), and were in Robson Group 5 (Table 1).

**Table 1:** Characteristics of term births by gestational age in Brazil, 2012–2019 (17,081,685)

Variables	**37 and 38 weeks**		39–41 weeks	
	N	%	N	%
**Maternal age (years)**				
14–19	850,216	13.91	1,927,452	17.57
20–34	4,236,326	69.29	7,736,809	70.54
35–49	1,027,458	16.81	1,303,424	11.88
**Marital status**				
Married/ Civil partnership	3,640,617	60.01	5,915,818	54.39
Single	2,329,020	38.39	4,822,248	44.34
Widow	11,550	0.19	18,424	0.17
Divorced	85,511	1.41	119,908	1.10
**Maternal education (years)**				
None	24,028	0.40	53,892	0.50
1–3	128,171	2.12	281,047	2.59
4–7	911,495	15.06	1,995,877	18.39
8–12	3,489,541	57.65	6,720,211	61.93
12 +	1,500,087	24.78	1,800,365	16.59
**Maternal ethnicity**				
White	2,628,221	44.07	3,842,312	35.94
Black	314,990	5.28	643,998	6.02
Asian	27,215	0.46	43,448	0.41
Mixed-race	2,952,097	49.50	6,073,132	56.80
Indigenous	40,770	0.68	3,842,312	0.83
**Number of prenatal visits**				
None	76,268	1.25	149,537	1.37
1–3	318,912	5.24	586,734	5.37
4–6	1,357,776	22.32	2,377,916	21.78
7+	4,330,738	71.19	149,537	71.47
**Newborns` sex**				
Male	3,167,327	37.62	5,543,573	50.55
Female	2,945,934	62.38	5,423,211	49.45
**Geographic region**				
North	539,265	8.82	1,133,252	10.33
Northeast	1,328,210	21.72	2,816,163	25.68
Southeast	2,771,724	45.33	4,585,251	41.81
South	938,409	15.35	1,559,013	14.21
Central-West	536,192	8.77	874,006	7.97
**Robson groups**				
Robson 1	1,017,345	16.64	2,143,650	19.55
Robson 2	1,148,225	18.78	2,031,345	18.52
Robson 3	1,118,057	18.29	2,483,349	22.64
Robson 4	703,770	11.51	1,337,167	12.19
Robson 5	1,762,676	28.83	2,631,709	24.00
Robson 6	92,468	1.51	119,278	1.09
Robson 7	124,135	2.03	162,718	1.48
Robson 8	134,964	2.21	38,771	0.35
Robson 9	12,360	0.20	19,698	0.18

CS: Cesarean section; Robson groups: 1 (Nulliparous women with a single cephalic pregnancy, ≥ 37 weeks gestation in spontaneous labor); 2 (Nulliparous women with single cephalic pregnancy, ≥ 37 weeks gestation who either had labor induced or were delivered by cesarean section before labor); 3 (Multiparous women without a previous uterine scar, with a single cephalic pregnancy, > 37 weeks gestation, in spontaneous labor); 4 (Multiparous women without a previous uterine scar, with single cephalic pregnancy, ≥ 37 weeks gestation who either had labor induced or were delivered by cesarean section before labor); 5 (All multiparous women with at least one previous CS, with a single cephalic pregnancy, ≥ 37 weeks gestation); 6 (All nulliparous women with a single breech pregnancy); 7 (All multiparous women with a single breech pregnancy, including women with a previous CS); 8 (All women with multiple pregnancies, including women with a previous CS); 9 (All women with a single pregnancy with a transverse or oblique lie, including women with previous CS(s).

The proportion of CS deliveries in the general population (Table 2) was higher among older mothers, married/in a civil partnership, were white, with a higher level of education, attended more prenatal visits, and lived in wealthier geographic regions (Table 2). The proportion of live births via CS varied according to the Robson groups, from 17.49% in Group 3, to 97.21% in Group 9 (Table S2). Following PSM, women who delivered via a CS had very similar characteristics to women who had delivered vaginally in the general population (Table S2). The distribution of scores is displayed in Figure S1.

**Table 2:** Characteristics of term births by type of delivery in Brazil, 2012–2019 (17,081,685)

Variables	Vaginal delivery		CS	
	N (7,619,183)	% (44.60)	N (9,462,502)	% (55.40)
**Maternal age (years old)**				
14–19	1,711,363	22.46	1,066,305	11.27
20–34	5,191,928	68.14	6,781,207	71.66
35–49	715,892	9.40	1,614,990	17.07
**Marital status**				
Married/ Civil partnership	3,710,614	49.15	5,845,821	62.23
Single	3,758,569	49.79	3,392,699	36.11
Widow	12,380	0.16	17,594	0.19
Divorced	67,303	0.89	138,116	1.47
**Maternal education (years)**				
None	57,292	0.76	20,628	0.22
1–3	263,882	3.50	145,336	1.55
4–7	1,735,882	23.05	1,171,490	12.50
8–12	4,788,937	63.59	5,420,815	57.83
12 + year	685,209	9.10	2,615,243	27.90
**Maternal ethnicity (years)**				
White	2,190,329	29.52	4,280,204	46.35
Black	501,537	29.52	457,451	4.95
Asian	30,695	0.41	39,968	0.43
Mixed-race	4,593,923	61.91	4,431,306	47.99
Indigenous	104,029	1.40	25,469	0.28
**Number of prenatal visits**				
None	146,100	1.93	79,705	0.85
1–3	605,011	7.99	300,635	3.19
4–6	2,045,605	27.01	1,690,087	17.93
7+	4,776,213	63.07	7,356,498	78.04
**Newborns` sex**				
Male	3,827,049	50.23	4,883,851	51.62
Female	3,791,490	49.77	4,577,655	48.38
**Geographic region**				
North	893,178	11.72	779,539	8.24
Northeast	2,120,736	27.83	2,023,637	21.39
Southeast	3,010,994	40.31	4,285,981	45.29
South	994,422	13.05	1,503,000	15.88
Central-West	539,853	7.09	870,345	9.20

CS: Cesarean Section

Early-term prevalence was 37.62% among those born by vaginal delivery, and 62.38% by CS (Table 3). Early-term births prevalence varied between Robson groups and type of delivery. Robson Groups 6 to 9 had a higher prevalence of early-term births, exceeding 88% among those born by CS. In Robson group 5, higher early term prevalence was also found in CS births (85.53%In the Robson Groups 1 to4, the prevalence of early-term birth by CS was higher in Robson Groups 2 (75.09%) and 4 (53.04%) than group 1 and 3 (Table 3).

**Table 3:** Early-term births by type of delivery according to the Robson Groups classification in Brazil, 2012–2019 (n = 17,081,68)

Robson Groups	Early-term births			PSM^*^		PAF	
	Total population	Vaginal	CS				
	N (%)	N (%)	N (%)	OR	CI 95%	%	CI 95%
**1**	1,017,345 (32.18)	554,043 (54.46)	463,302 (45.54)	1.04	1.04–1.05	2.83	2.45–3.20
**2**	1,148,225 (36,11)	286,022 (24.91)	862,203 (75.09)	1.50	1.49–1.51	23.69	23.30–24.00
**3**	1,118,057 (31.05)	891,385 (79.73)	226,672 (20.27)	1.30	1.29–1.31	16.10	15.70-16.56
**4**	703,770 (34.48)	330,500 (46.96)	373,270 (53.04)	1.57	1.56–1.58	25.75	25.40-26.09
**5**	1,762,676 (40.11)	202,212 (11.47)	1,560,464 (88,53)	1.36	1.35–1.37	18.29	17.89–18.69
**6**	92,468 (43.67)	6,248 (6.76)	86,220 (93.24)	1.16	1.11–1.21	8.47	5.99–10.89
**7**	124,135 (43.27)	13,674 (11.02)	110,461 (88.98)	1.19	1.16–1.23	10.02	8.35–11.65
**8**	132,964 (77.68)	15,857 (11.75)	119,107 (88.25)	1.46	1.40–1.52	10.65	9.58–11.71
**9**	12,360 (38.56)	311 (2.52)	12,049 (97.48)	1.09	0.89–1.32	5.45	-7.68-16.58
**All groups**	6,114,000 (37.79)	2,300,252 (37.62)	3,813,748 (62.38)	1.32	1.32–1.32	17.00	16.87–17.14

The early-term birth (37 and 38 weeks gestation) was compared with births at 39 to 41 weeks gestation. OR (odds ratio) from a logistic regression in which vaginal deliveries are the comparison group. *Variables used in PSM (propensity score matching): race/ethnicity, maternal education, marital status, number of prenatal appointments, maternal age at delivery, newborns` sex and year of birth. CS: Cesarean section. PAF: population attributable fraction. Robson groups: 1 (Nulliparous women with a single cephalic pregnancy, ≥ 37 weeks gestation in spontaneous labor); 2 (Nulliparous women with single cephalic pregnancy, ≥ 37 weeks gestation who either had labor induced or were delivered by cesarean section before labor); 3 (Multiparous women without a previous uterine scar, with a single cephalic pregnancy, > 37 weeks gestation, in spontaneous labor); 4 (Multiparous women without a previous uterine scar, with single cephalic pregnancy, ≥ 37 weeks gestation who either had labor induced or were delivered by cesarean section before labor); 5 (All multiparous women with at least one previous CS, with a single cephalic pregnancy, ≥ 37 weeks gestation); 6 (All nulliparous women with a single breech pregnancy); 7 (All multiparous women with a single breech pregnancy, including women with a previous CS); 8 (All women with multiple pregnancies, including women with a previous CS); 9 (All women with a single pregnancy with a transverse or oblique lie, including women with previous CS(s).

Following adjustment via PSM, the odds of an early-term birth was 32% (95% CI 1.32–1.32) higher among live births delivered by CS than those born by vaginal delivery in all Robson groups. The odds for an early-term birth varied between Robson groups. When compared to those born by vaginal delivery, the odds of an early-term birth were more likely in those born by CS among nulliparous (Group 2 - OR 1.50; 95% CI 1.49–1.51) and multiparous women (Robson group 4 - OR 1.57; 95% CI 1.56–1.58) without a previous CS, with a single cephalic pregnancy, at term, and who had their labor induced or a pre-labor CS. Increased odds of an early-term birth were also observed among live births of multiparous women without a previous CS, with a single cephalic pregnancy, at term, and in spontaneous labor (Robson Group 3 - OR 1.30; 95% CI 1.29–1.31). The odds of an early-term birth were similar in those born by CS than those born by vaginal delivery in the group of nulliparous women with a single cephalic pregnancy, at term, and in spontaneous labor (Robson Group 1 - OR 1.04; 95% CI 1.04–1.05) (Table 3).

The live births from multiparous women with at least one previous CS and with a single cephalic pregnancy (Robson Group 5), delivered by CS, showed higher odds of being early-term than those delivered by vaginal delivery (OR 1.36; 95% CI 1.35–1.37). In addition, infants born by CS were more likely to be early-term births when born to nulliparous or multiparous women with a single breech pregnancy (Robson Group 6 - OR 1.16; 95% CI 1.11–1.21) and (Robson Group 7 - OR 1.19; 95% CI 1.16–1.23, respectively). Higher odds of early-term birth were also observed among live births of women with multiple pregnancies (Robson Group 8 - OR 1.46; 95% CI 1.40–1.52) (Table 3).

Attributable fraction analysis indicated that 17% (95% CI 16.87–17.1) of early-term births were attributed to CSs, with a marked variation between Robson groups and a higher PAF in Groups 2 and 4, 23.69% (95% CI 23.30–24.00) and 25.75% (95% CI 25.40-26.09), respectively (Table 3). Additional analyses showed a higher prevalence of early-term births at 38 weeks of gestation, when compared to those at 37 weeks (27.95% versus 14.50%). For almost all Robson groups, the magnitude of the association between CSs and early-term birth was greater among those born at 38 weeks of gestation, compared to those born at 37 weeks (Tables S3 and S4).

In the analysis by geographic regions, we observed a greater odd of early-term birth among those born by CS in the South (OR 1.49; 95% CI 1.48–1.50), Central-West (OR 1.34; 95% CI 1.33–1.35) and Southeast (OR 1.33; 95% CI 1.32–1.33). In the poorest regions of the country, these odds were 1.21 (95% CI 1.20–1.22) in the North and 1.19 (95% CI 1.18–1.19) in the Northeast. Analyzes for the different Robson Groups also show high chances of early-term birth among CS born in Groups 2 and 4 in all regions of the country, especially in the South and Central-West regions (Table S5).

The robustness analyses with a finer caliper in the PSM model, and bivariate and multivariate logistic regression produced similar results to the primary analyses (PSM) (Table S6).

## Discussion

In this population-based study with more than 17 million live births, we observed that a cesarean delivery was associated with a more than 1.3-fold increase in the odds of an early-term birth, compared to a vaginal delivery. In the stratified analysis by Robson classification, we observed that among the live births from women in Robson Groups 2 and 4, those born by CS had a 50%, or greater, increase in the odds of an early-term birth, compared to those born by vaginal delivery. Increased odds of an early-term birth were also found among those born by CS in Robson Groups 3, 5, and 6–8, ranging from 16 to 46%. In addition, we observed greater odds of early-term birth among those born by CS in the South, Central-West and Southeast regions, especially in Robson Groups 2 and 4.

The prevalence of early-term births was higher in live births by CS than in those by vaginal delivery. We found a high proportion of early-term births from older, white, and higher educated mothers. The prevalence of early-term births (35.79%) was higher than those previously reported in developed countries, such as Japan (30.8%), Malta (30.7%), and Luxembourg (29.7%) [17]. However, the high prevalence of early-term births was similar to that observed in local studies [11, 25]. Early-term births accounted for 35% (95% CI 33.4%-36.7%) of all live births in the “Birth in Brazil study” [11]. Raspantini et al. (2016) observed that early-term births were responsible for more than 34.5% of births in the city of São Paulo [25].

Very few studies have evaluated the odds of an early-term birth associated with CSs [8–10]. A Brazilian study showed that early-term births were 1.64 (95% CI 1.62–1.61) times higher in municipalities with very high ones (≥ 80%), when compared to municipalities with lower CS rates (< 30%) [8]. The association between CSs and early term delivery may differ between women with maternal or foetal indications (e.g., preeclampsia, placental abruption, and foetal distress), and no medical indication. There is insufficient information to identify high-risk pregnancies on SINASC data and, therefore, we used the Robson classification to evaluate a CS indication proxy, through which we were able to stratify groups of births of women with expected lower and higher CS rates and needs.

Our results show an increased risk of an early-term birth among those born by CS from nulliparous (Group 2) and multiparous women (Group 4). The high CS rates found in Robson Groups 2 and 4 may be related to the number of women undergoing a CS before the onset of labor (Groups 2b and 4b), which was higher than those undergoing induction (Groups 2a and 4a). Our hypothesis is that, in part, these groups had CSs due to medical reasons, or maternal preference, which may contribute to the increase in early-term births in these groups. A previous Brazilian study reinforces our hypothesis. Leal et al. (2017) [11] observed that among early-term live births, 47% were provider-initiated, mainly pre-labor CSs, and 30% were provider-initiated in women without clinical or obstetric needs.

Our hypothesis is corroborated by a high prevalence of CSs among highly educated women who live in richer geographic regions of the country. In addition, disparities in access to well-indicated CSs among women in the North and Northeast may also explain our findings, as well observed as in other Brazilian studies [26–29]. That also refers to a high number of CSs performed on women attended by private health services [26]. Raspantini et al. (2016) [25] observed a higher proportion of early-term births (52.20%) in private than in public network hospitals (30.30%), which may explain the gestational age mean of 38 weeks in private hospitals, compared to 39 weeks in public hospitals. Similarly, Diniz et al. (2016) [30] observed that the highest concentration of cesarean births in the private system occurred early-term (35%). It is known that performing an elective CS may lead to short- and long-term health effects for children [7]. Thus, public policies should be directed at reducing the number of elective CSs, especially in the private health sector.

Our results also showed an increased odds of an early-term birth among those born througha CS in the multiparous group without a previous CS (Robson Group 3). These results may be related to maternal preference for a CS for tubal ligation, as mentioned by Domingues et al. (2014) [2]. We also observed increased odds of an early-term birth among live births due to a CS in multiparous women with a previous CS (Robson Group 5). Group 5 represents the largest group of live births in this study (over 25%), and has one of the highest CS rates observed (over 85%). In a previous study, women with repeated elective CSs more frequently gave birth at the beginning of term [9, 10]. Concern about an increased risk of uterine rupture among women with multiple CS scars, following the onset of spontaneous labor may explain the high rates of early elective Cs in these groups [9].

In this study, we identified increased odds of early-term birth associated with CS among those born to nulliparous or multiparous women with a single breach pregnancy (Groups 6 and 7, respectively) and multiple pregnancies (Group 8). Studies have shown that there is an increased risk of perinatal mortality and morbidity when a twin pregnancy continues beyond 37 weeks, and that lower risks are seen when twins are born between 36 and 38 weeks [31, 32]. Our study findings of our study may indicate the higher indication (preference) of a CS as a type of delivery for multiple pregnancies, although the safest method for delivering twins at term, or close to term, remains a controversial issue [33–35].

A CS is an effective intervention to protect maternal and fetal health when performed for well-defined clinical reasons [6]. A CS may be the consequence of a situation that may be identified during pregnancy (Groups 6 or above), or result from a cascade of unexpected and unpredictable events (eclampsia, HELLP syndrome, fetal asphyxia, and uterine rupture) [36] in women not classified in Groups 6–9. A high cesarean section rate is expected in Robson Groups 6 to 9, and consequent early delivery results from appropriate clinical decisions. CSs in the low-risk group may result from unexpected developments requiring a CS. In both groups, it may result from non-clinical factors, such as convenience for obstetricians or mothers [26, 27].

Our study showed that 17% of Brazilian early-term births were attributed to CSs. We also found higher population attributable risks to CSs in live births to mothers in Robsons Groups 2 and 4, in which low CS rates are expected. Our results suggest that Brazil faces two interrelated epidemics: a CS epidemic and one of early-term births, especially among those born at 38 gestational weeks, related to high CS rates in women with induced labor and before labor (Robson Groups 2 and 4). It is known that early-term infants are at a greater risk of adverse birth outcomes than those born at full-term [11–15], especially when born by CS [12]. Thus, different organizations, such as the American College of Obstetricians and Gynecologists, have recommended that CSs without clinical indications should not be performed before the 39th week of gestation [37].

Although CS rates are still high in Brazil, different programs, policies and strategies created and implemented in health services aim to improve the quality of obstetric care, especially during labor and birth [3–5]. In this scenario, the collaborative obstetric care model is identified, with integration of doctors and obstetric nurses into the team, in addition to other professionals, such as doulas, aiming to reduce the use of unnecessary interventions and CSs [38]. Thus, there is a need for changes in the predominant and traditional model of obstetric care in Brazil, with improvements to the quality of prenatal care and childbirth and reductions in adverse outcomes for mothers and children.

### Strengths and Limitations

This study has several strengths. To the best of our knowledge, this is the first study to use the Robson classification to assess CSs and early-term births. The Robson classification helps to identify target groups that may benefit from health implementations or interventions that contribute towards the reduction of CS rates in Brazil. The use of PSM made it possible to reduce the effect of confounding due to socioeconomic differences between live births of the women who had a vaginal delivery or CS. We also studied and compared early-term births by Robson subgroups and geographic areas of the country, rather than focusing on a small subset. However, there are also a number of limitations to this study. The main limitation is the lack of data on maternal and fetal health conditions (e.g., preeclampsia and fetal distress). However, the Robson classification proved to be a viable proxy to assess the clinical indication of a CS through groups with the lowest and highest expected rates and clinical needs for CSs. Further limitation is the high percentage of missing data for some of the main variables used to classify women into one of the Robson Groups, and possible errors in identifying the onset labor. In addition, we do not know if the women went into labor and there was a failure in conducting the birth by professionals, with the use of obstetric interventions, which culminated in the evolution of CSs. In addition, residual confounding is possible, due to the lack of data on maternal comorbidities (e.g., diabetes and, hypertension), pregnancy body mass index, and the quality of local health services.

## Conclusions

Our results provide evidence that CSs are associated with high odds of early-term births, with the highest observed among those born by CS in Robson groups 2 and 4. The association between cesarean delivery and early-term birth were also observed among live births of women in Group 3, 5, and 6 to 8. The results of this study provide important evidence for the implementation of public policies targeting the reduction of unnecessary CSs, especially the Robson groups with a low indication, which will consequently allow a reduction in the number of early-term births. We emphasize the importance of conducting further studies that focus on providing additional knowledge of early-term births, especially in countries with high CS rates.

### Electronic supplementary material

Below is the link to the electronic supplementary material.

Supplementary Material 1

## Data Availability

The data described in the manuscript, code book and analytical code will be made available upon request to the corresponding author, E-mail: linny_Rochaa@hotmail.com. Importantly, restrictions apply to the availability of these data. However, upon reasonable request and provided all ethical and legal requirements are met, the institutional data curation team can make the data available. Further information can be obtained at https://cidacs.bahia.fiocruz.br/acesso-aos-dados/.

## List of abbreviations

CS: Cesarean section
CI: Confidence interval
CIDACS: Centre for Data and Knowledge Integration for Health
CIDACS-RL: Centre for Data and Knowledge Integration for Health - Record Linkage
ISC-UFBA: Federal University of Bahia’s Collective Health Institute
LMICs: low- and middle-income countries
NICU: Neonatal Intensive Care Units
OR: Odds Ratio
PAF: population attributable fraction
PSM: propensity score matching
SINASC: National live birth system
HELLP: hemolysis, elevated liver enzymes and low platelets
