# Supplementary material for: Cesarean sections and early-term births according to Robson classification: a population-based study with more than 17 million births in Brazil
Source: BMC Pregnancy Childbirth. 2023 Aug 3;23:562. doi: 10.1186/s12884-023-05807-y (PMC10399022; doi:10.1186/s12884-023-05807-y)
Supplement: Supplementary file 1 — Supplementary Material 1 [file 12884_2023_5807_MOESM1_ESM.doc]

**Supplementary material**

**Cesarean sections and early-term births according to Robson classification: a population-based study with more than 17 million births in Brazil**

| **Table S1. Robson classification and cesarean section rate in Brazil, 2012-2019 (n=17,081,685)** | | | | | | | |
| --- | --- | --- | --- | --- | --- | --- | --- |
| **Robson Classification** | | Number of women in group | Relative group size to overall facility population^1^ (%) | Number of CS in each group | CS rate in each group^2^ (%) | Absolute group contribution to overall CS rate^3^ (%) | Relative contribution of each of the 10 groups to overall CS rate^4^ (%) |
| **1** | Nulliparous women with a single cephalic pregnancy, ≥37 weeks gestation in spontaneous labor | 3,160,995 | 18.51 | 1,372,242 | 43.41 | 8.03 | 14.50 |
| **2** | Nulliparous women with single cephalic pregnancy, ≥37 weeks gestation who either had labor induced or were delivered by cesarean section before labor | 3,179,570 | 18.61 | 2,193,873 | 69.00 | 12.84 | 23.18 |
| **3** | Multiparous women without a previous uterine scar, with a single cephalic pregnancy, >37 weeks gestation, in spontaneous labor | 3,601,406 | 21.08 | 630,008 | 17.49 | 3.69 | 6.66 |
| **4** | Multiparous women without a previous uterine scar, with single cephalic pregnancy, ≥37 weeks gestation who either had labor induced or were delivered by cesarean section before labor | 2,040,937 | 11.95 | 903,935 | 44.29 | 5.29 | 9.55 |
| **5** | All multiparous women with at least one previous CS, with a single cephalic pregnancy, ≥37 weeks gestation | 4,394,385 | 25.73 | 3,735,942 | 85.02 | 21.87 | 39.48 |
| **6** | All nulliparous women with a single breech pregnancy | 211,746 | 1.24 | 195,561 | 92.36 | 1.14 | 2.07 |
| **7** | All multiparous women with a single breech pregnancy, including women with a previous CS | 286,853 | 1.68 | 250,154 | 87.21 | 1.46 | 2.64 |
| **8** | All women with multiple pregnancies, including women with a previous CS | 173,735 | 1.02 | 149,625 | 86.12 | 0.88 | 1.58 |
| **9** | All women with a single pregnancy with a transverse or oblique lie, including women with previous CS(s) | 32,058 | 0.19 | 31,162 | 97.21 | 0.18 | 0.33 |
| **10** | All women with a single cephalic pregnancy <37 weeks gestation, including women with previous CS(s) | 0 | 0.00 | 0 | 0.00 | 0.00 | 0.00 |
| **Total*** | | 17.081.685 | 100 | 9.462.502 | 622.11 | 55.40 | 100.00 |
| CS: Cesarean section.  * These totals and percentages come from the data in the table  1. Group size (%) = n of women in the group / total N women delivered in the hospital x 100  2. Group CS rate (%) = n of CS in the group / total N of women in the group x 100  3. Absolute contribution (%) = n of CS in the group / total N of women delivered in the hospital x 100  4. Relative contribution (%) = n of CS in the group / total N of CS in the hospital x 100 | | | | | | | |

| **Table S2. Standardized mean differences (SMD) before and after matching for the matching covariates, for all Robson groups (n=17,081,685)** | | | | | | | | |
| --- | --- | --- | --- | --- | --- | --- | --- | --- |
|  | **Before matching** | | | | **After matching** | | | |
|  | Vaginal delivery | Cesarean section | |  | Vaginal delivery | Cesarean section | |  |
|  | N (%) | N (%) | SMD | | N (%) | N (%) | SMD | |
| **Maternal age (years old)** |  |  |  | |  |  |  | |
| 14-19 | 1,711,363 (22.5) | 1066305 (11.3) | 0.353 | | 1,009,824 (17.1) | 1014599 (17.2) | 0.025 | |
| 21-34 | 5,191,928 (68.1) | 6781207 (71.7) |  | | 4,284,614 (72.4) | 1,235,686 (71.6) |  | |
| 35-49 | 715,892 (9.4) | 1614990 (17.1) |  | | 619,657 (10.5) | 663811 (11.2) |  | |
| **Marital status** |  |  |  | |  |  |  | |
| Married/Civil partnership | 3,710,614 (49.2) | 5845821 (62.2) | 0.280 | | 31100409 (52.6) | 2,958,705 (50.0) | 0.053 | |
| Single | 3,758,569 (49.8) | 3392699 (36.1) |  | | 2731024 (46.2) | 2,870,148 (48.5) |  | |
| Widow | 12,380 (0.2) | 17594 (0.2) |  | | 10127 (0.2) | 13,014 (0.2) |  | |
| Divorced | 67,303 (0.9) | 138116 (1.5) |  | | 62536 (1.1) | 72,229 (1.2) |  | |
| **Maternal education (years)** | |  |  | |  |  |  | |
| None | 57,292 (0.8) | 20628 (0.2) | 0.549 | | 19,614 (0.3) | 19,705 (0.3) | 0.260 | |
| 1-3 | 263,882 (3.5) | 145336 (1.6) |  | | 138,401 (2.3) | 139,135 (2.4) |  | |
| 4-7 | 1,736,882 (23.0) | 1171490 (12.5) |  | | 1,096,728 (18.5) | 1,121,363 (19.0) |  | |
| 8-12 | 4,788,937 (63.6) | 5420815 (57.8) |  | | 3,998,447 (67.6) | 4,377,776 (74.0) |  | |
| 12 + | 685,209 (9.1) | 2615243 (27.9) |  | | 660,876 (11.2) | 256,117 (4.3) |  | |
| **Maternal ethnicity** |  |  |  | |  |  |  | |
| White | 2,190,329 (29.5) | 4280204 (46.4) | 0.368 | | 2025379 (34.2) | 1791902 (30.3) | 0.085 | |
| Black | 501,537 (6.8) | 457451 (5.0) |  | | 366536 (6.2) | 401641 (6.8) |  | |
| Asian | 30,695 (0.4) | 39968 (0.4) |  | | 24307 (0.4) | 26999 (0.5) |  | |
| Mixed-race | 4,593,923 (61.9) | 4431306 (48.0) |  | | 3473667 (58.7) | 3668789 (62.0) |  | |
| Indigenous | 104,029 (1.4) | 25469 (0.3) |  | | 24207 (0.4) | 24770 (0.4) |  | |
| **Number of prenatal visits** | | | | | | | | |
| None | 146,100 (1.9) | 79705 (0.8) | 0.349 | | 60772 (1.0) | 68291 (1.2) | 0.028 | |
| 1-3 | 605,011 (8.0) | 300635 (3.2) |  | | 274574 (4.6) | 281973 (4.8) |  | |
| 4-6 | 2,045,605 (27.0) | 1690087 (17.9) |  | | 1383237 (70.9) | 1441145 (24.4) |  | |
| 7+ | 4,776,213 (63.1) | 7356498 (78.0) |  | | 4195513 (70.9) | 4122687 (69.7) |  | |
| **Newborns` sex** |  |  |  | |  |  |  | |
| Male | 3, 827,049(50.2) | 4883851 (51.6) | 0.028 | | 3017322 (51.0) | 2959345 (50.0) | 0.020 | |
| Female | 3,791,490 (49.8) | 4577655 (48.4) |  | | 2896774 (49.0) | 2954751 (50.0) |  | |
| **Year of birth** |  |  |  | |  |  |  | |
| 2012 | 825,082 (10.8) | 1019057 (10.9) | 0.029 | | 623239 (10.5) | 623774 (10.5) | 0.030 | |
| 2013 | 843,682 (11.1) | 1074242 (11.4) |  | | 641286 (10.8) | 661009 (11.2) |  | |
| 2014 | 931,500 (12.2) | 1233117 (13.0) |  | | 737552 (12.5) | 735810 (12.4) |  | |
| 2015 | 966,940 (12.7) | 1158241 (12.2) |  | | 731342 (12.4) | 719959 (12.2) |  | |
| 2016 | 1,011,752 (13.3) | 1229075 (13.0) |  | | 775548 (13.1) | 731867 (12.4) |  | |
| 2017 | 993,443 (13.0) | 1213686 (12.8) |  | | 770382 (13.0) | 765693 (12.9) |  | |
| 2018 | 1,017,881 (13.4) | 1255850 (13.3) |  | | 805092 (13.6) | 842853 (14.3) |  | |
| 2019 | 1,028,903 (13.5) | 1279234 (13.5) |  | | 829655 (14.0) | 833131 (14.1) |  | |

**
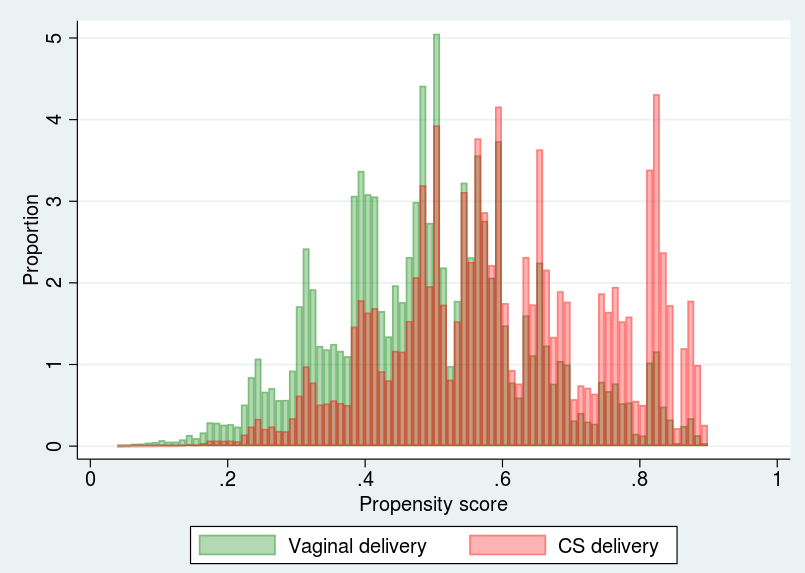
**

**Figure S1:** Propensity score distribution by type of delivery for the entire study population.

**CS**: Cesarean section

| **Table S3. Additional analyses for early-term births (37 weeks gestation) by type of delivery according to the Robson classification in Brazil, 2012-2019 (n=12,827,121).** | | | | | | | | | | | |
| --- | --- | --- | --- | --- | --- | --- | --- | --- | --- | --- | --- |
| **Robson Groups** | **Early-term births** | | | **Unadjusted analysis** | | **Adjusted analysis*** | | **PSM**** | | **PAF** | |
|  | **Total population** | **Vaginal** | **CS** |  |  |  |  |  |  |  |  |
|  | **N (%)** | **N (%)** | **N (%)** | **OR** | **CI 95%** | **OR** | **CI 95%** | **OR** | **CI 95%** | **%** | **CI 95%** |
| **1** | 316,470 (12,86) | 179,109 (56.60) | 137,361 (43.40) | 1.04 | 1.03-1.05 | 1.00 | 1.00-1.01 | 0.98 | 0.97-0.99 | -1.85 | -2.66 – -1.05 |
| **2** | 322,042 (13,68) | 91,304 (28.35) | 230,738 (71,65) | 1.33 | 1.32-1.34 | 1.21 | 1.22-1.23 | 1.31 | 1.29-1.32 | 20.70 | 20.02-21.36 |
| **3** | 354,856 (12,50) | 283,750 (79,96) | 71,106 (20.04) | 1.29 | 1.28-1.30 | 1.30 | 1.29-1.31 | 1.30 | 1.29-1.32 | 20.49 | 19.66-21.31 |
| **4** | 128,018 (14,02) | 107,181 (49.16) | 110,837 (50.84) | 1.57 | 1.56-1.59 | 1.49 | 1.47-1.51 | 1.49 | 1.47-1.50 | 28.93 | 28.26-29.59 |
| **5** | 494,577 (15,82) | 63,427 (12.82) | 431,150 (87,18) | 1.43 | 1.41-1.44 | 1.30 | 1.29-1.31 | 1.26 | 1.24-1.27 | 18.00 | 17.2-18.90 |
| **6** | 29,859 (20,02) | 2,135 (7.15) | 27,729 (92.85) | 1.18 | 1.12-1.24 | 1.12 | 1.06-1.18 | 1.12 | 1.05-1.20 | 8.89 | 3.38-13.69 |
| **7** | 40,597 (19,97) | 4,764 (11.74) | 35,832 (88,26) | 1.24 | 1.20-1.28 | 1.14 | 1.10-1.18 | 1.16 | 1.11-1.21 | 11.24 | 7.95-14.42 |
| **8** | 79,282 (67,16) | 8,563 (10,80) | 70,719 (89,20) | 2.23 | 2.16-2.31 | 1.58 | 1.53-1.64 | 1.54 | 1.47-1.61 | 17.03 | 15.41-18.61 |
| **9** | 3,731 (15,92) | 104 (2,79) | 3,627 (97,21) | 1.05 | 0.86-1.32 | 1.03 | 0.82-1.28 | 0.94 | 0.69-1.27 | -5,88 | -37,79-18,63 |
|  |  |  |  |  |  |  |  |  |  |  |  |
| **All groups** | 1,849,436 (14,50) | 740,337 (39,82) | 1,119,099 (60,18) | 1.42 | 1.42-1.43 | 1.33 | 1.32-1.33 | 1.28 | 1.27-1.28 | 19.03 | 18.76-19.30 |
| CS: Cesarean section; PAF: population attributable fraction; The early-term birth (37 weeks gestation) was compared with births at 39 to 41 weeks gestation; OR (odds ratio) from a logistic regression in which vaginal deliveries are the comparison group; * adjusted by PSM (propensity score matching) covariates: race/ethnicity, maternal education, marital status, number of prenatal appointments, maternal age at delivery, newborns` sex and year of birth ;** 0·1 width caliper; Robson groups: 1 (Nulliparous women with a single cephalic pregnancy, ≥ 37 weeks gestation in spontaneous labor); 2 (Nulliparous women with single cephalic pregnancy, ≥ 37 weeks gestation who either had labor induced or were delivered by cesarean section before labor); 3 (Multiparous women without a previous uterine scar, with a single cephalic pregnancy, > 37 weeks gestation, in spontaneous labor); 4 (Multiparous women without a previous uterine scar, with single cephalic pregnancy, ≥ 37 weeks gestation who either had labor induced or were delivered by cesarean section before labor); 5 (All multiparous women with at least one previous CS, with a single cephalic pregnancy, ≥ 37 weeks gestation); 6 (All nulliparous women with a single breech pregnancy); 7 (All multiparous women with a single breech pregnancy, including women with a previous CS); 8 (All women with multiple pregnancies, including women with a previous CS); 9 (All women with a single pregnancy with a transverse or oblique lie, including women with previous CS(s). | | | | | | | | | | | |
|  | | | | | | | | | |  |  |

| **Table S4. Additional analyses for early-term births (38 weeks gestation) by type of delivery according to the Robson classification in Brazil, 2012-2019 (n=15,222,249)** | | | | | | | | | | | |
| --- | --- | --- | --- | --- | --- | --- | --- | --- | --- | --- | --- |
| **Robson Groups** | **Early-term births** | | | **Unadjusted analysis** | | **Adjusted analysis** | | **PSM**** | | **PAF** | |
|  | **Total population** | **Vaginal** | **CS** |  |  |  |  |  |  |  |  |
|  | **N (%)** | **N (%)** | **N (%)** | **OR** | **CI 95%** | **OR** | **CI 95%** | **OR** | **CI 95%** | **%** | **CI 95%** |
| **1** | 700,875 (24.64) | 374,934 (53.50) | 325,941 (46.50) | 1.18 | 1.17-1.19 | 1.10 | 1.09-1.10 | 1.07 | 1.06-1.09 | 5.15 | 4.69-5.61 |
| **2** | 826,183 (28.91) | 194,718 (23.57) | 631,465 (76.43) | 1.70 | 1.69-1.71 | 1.44 | 1.43-1.45 | 1.59 | 1.58-1.60 | 29.11 | 28.75-29.48 |
| **3** | 763,201 (23,51) | 607,635 (79.62) | 155,566 (20.38) | 1.32 | 1.31-1.33 | 1.28 | 1.28-1.29 | 1.30 | 1.29-1.31 | 17.78 | 17.24-18.31 |
| **4** | 485,752 (26.65) | 223,319 (45.97) | 262,433 (54.03) | 1.79 | 1.77-1.80 | 1.62 | 1.61-1.64 | 1.61 | 1.60-1.63 | 29.68 | 29.26-30.08 |
| **5** | 1,268,099 (32.52) | 138,785 (10.94) | 1,129,314 (89.06) | 1.71 | 1.70-1.72 | 1.45 | 1.11-1.46 | 1.40 | 1.39-1.42 | 22.06 | 21.59-22.55 |
| **6** | 62,604 (34.42) | 4,113 (6.57) | 58,491 (93.43) | 1.29 | 1.24-1.34 | 1.15 | 1.11-1.20 | 1.18 | 1.12-1.24 | 10.80 | 7.59-13.92 |
| **7** | 83,539 (33.92) | 8,910 (10.67) | 74,629 (89.33) | 1.38 | 1.35-1.42 | 1.21 | 1.18-1.25 | 1.21 | 1.17-1.25 | 12.41 | 10.25-14.53 |
| **8** | 55,682 (58.95) | 7,294 (13.10) | 48,388 (86.90) | 1.79 | 1.73-1.86 | 1.43 | 1.38-1.48 | 1.37 | 1.31-1.44 | 14.18 | 12.19-16.13 |
| **9** | 8,629 (30.46) | 207 (2.40) | 8,422 (97.60) | 1.25 | 1.06-1.46 | 1.20 | 1.02-1.42 | 1.16 | 0.93-1.46 | 10.45 | -5.60-2.41 |
|  |  |  |  |  |  |  |  |  |  |  |  |
| **All groups** | 4,254,564 (27.95) | 1,559,915 (36.66) | 2,694,649 (63.34) | 1.63 | 1.62 - 1.63 | 1.42 | 1.42-1.43 | 1.34 | 1.34-1.35 | 19.70 | 19.53-19.84 |
| CS: Cesarean section; PAF: population attributable fraction; The early-term birth (38 weeks gestation) was compared with births at 39 to 41 weeks gestation; OR (odds ratio) from a logistic regression in which vaginal deliveries are the comparison group; *adjusted by PSM covariates; ** 0·1 width caliper; Robson groups: 1 (Nulliparous women with a single cephalic pregnancy, ≥ 37 weeks gestation in spontaneous labor); 2 (Nulliparous women with single cephalic pregnancy, ≥ 37 weeks gestation who either had labor induced or were delivered by cesarean section before labor); 3 (Multiparous women without a previous uterine scar, with a single cephalic pregnancy, > 37 weeks gestation, in spontaneous labor); 4 (Multiparous women without a previous uterine scar, with single cephalic pregnancy, ≥ 37 weeks gestation who either had labor induced or were delivered by cesarean section before labor); 5 (All multiparous women with at least one previous CS, with a single cephalic pregnancy, ≥ 37 weeks gestation); 6 (All nulliparous women with a single breech pregnancy); 7 (All multiparous women with a single breech pregnancy, including women with a previous CS); 8 (All women with multiple pregnancies, including women with a previous CS); 9 (All women with a single pregnancy with a transverse or oblique lie, including women with previous CS(s). | | | | | | | | | | | |

**Table S5: Early term by mode of delivery according to Robson classification and** **geographic region**, **2012-2019.**

| **Robson Groups** | **Early term** | | **Propensity score matching^*^** | | **PAF** | | |
| --- | --- | --- | --- | --- | --- | --- | --- |
|  | **Vaginal** | **CD** |  |  |  |  |  |
|  | **N (%)** | **N (%)** | **OR** | **IC 95%** | **%** | IC 95% | |
| **North** | | | | | | | |
| **1** | 68,619 (59.91) | 45,927 (40.09) | 0.90 | 0.89-0.92 | -7.53% | -8.81% - | -6.27% |
| **2** | 13,797 (28.46) | 34,688 (71.54) | 1.33 | 1.29- 1.37 | 17.62% | 16.05% | 19.15% |
| **3** | 132,330 (80.75) | 31,556 (19.25) | 1.33 | 1.30-1.35 | 17.63% | 16.49% | 18.76% |
| **4** | 17,993 (45.26) | 21,765 (54.74) | 1.57 | 1.52- 1.61 | 26.51% | 25.04% | 27.94% |
| **5** | 24,768 (16.98) | 121,119 (83.02) | 1.18 | 1.16-1.21 | 10.63 | 9.33 | 11.91 |
| **6** | 473 (10.30) | 4,120 (89.70) | 1.08 | .93- 1.27 | 5.17% | -5.25% | 14.55% |
| **7** | 1,316 (12.10) | 9,563 (87.90) | 1.30 | 1.18-1.42 | 15.67% | 10.38% | 20.65% |
| **8** | 1,406 (14.48) | 8,302 (85.52) | 1.62 | 1.45-1.81 | 17.48% | 13.71% | 21.09% |
| **9** | 37 (2.15) | 1,686 (97.85) | 1.09 | 0.60- 1.99 | 5.56% | -38.37% | 35.54% |
| **Overall** | 260,739 (48.33) | 278,726 (51.67) | 1.21 | 1.20-1.22 | -7.53% | -8.81% | -6.27% |
| **Northeast** | | | | | | | |
| **1** | 169,480 (58.42) | 120,642 (41.58) | 0.93 | 0.92-0.94 | -5.34% | -6.14% | -4.55% |
| **2** | 51,348 (32.09) | 108,668 (67.91) | 1.34 | 1.32- 1.36 | 18.49% | 17.67% | 19.31% |
| **3** | 275,655 (78.88) | 73,827 (21.12) | 1.22 | 1.20-1.23 | 12.63% | 11.84% | 13.41% |
| **4** | 58,116 (47.50) | 64,238 (52.50) | 1.56 | 1.53-1.58 | 26.65% | 25.79% | 27.51% |
| **5** | 43,114 (12.96) | 289,505 (87.04) | 1.17 | 1.15-1.19 | 10.16 | 9.15% | 11.15% |
| **6** | 1,606 (10.01) | 14,434 (89.99) | 1.05 | 0.96-1.14 | 2.97% | -2.66% | 8.30% |
| **7** | 3,977 (15.81) | 21,174 (84.19) | 1.10 | 1.04-1.16 | 5.80% | 2.33% | 9.14% |
| **8** | 5,081 (17.02) | 24,771 (82.98) | 1.39 | 1.30-1.48 | 11.05% | 8.97% | 13.09% |
| **9** | 68 (2.64) | 2,506 (97.36) | 0.81 | 0.53- 1.24 | -15.09% | -53.06% | 13.46% |
| **Overall** | 608,445 (45.81) | 719,765(54.19) | 1.19 | 1.18-1.19 | 11.19% | 10.88% | 11.50% |
| **Southeast** | | | | | | | |
| **1** | 210,071 (53.46) | 182,899 (46.54) | 1.12 | 1.11-1.13 | 7.46% | 6.89% | 8.03% |
| **2** | 154,940 (24.52) | 476,939 (75.48) | 1.54 | 1.53-1.55 | 24.63% | 24.22% | 25.03% |
| **3** | 313,568 (81.14) | 72,868 (18.86) | 1.34 | 1.32-1.36 | 17.42% | 16.69% | 18.15% |
| **4** | 179,138 (48.85) | 187,604 (51.15) | 1.52 | 1.51- 1.54 | 23.60% | 23.12% | 24.07% |
| **5** | 87,968 (10.66) | 736,939 (89.34) | 1.44 | 1.42-1.45 | 20.94% | 20.37% | 21.51% |
| **6** | 2,631 (5.79) | 42,828 (94.21) | 1.21 | 1.12-1.30 | 10.15% | 6.45% | 13.70% |
| **7** | 5,321 (9.74) | 49,283 (90.26) | 1.18 | 1.12- 1.24 | 8.78% | 6.16% | 11.33% |
| **8** | 6,685 (10.43) | 57,421 (89.57) | 1.36 | 1.27-1.46 | 7.07% | 5.54% | 8.59% |
| **9** | 126 (2.73) | 4,495 (97.27) | 1.15 | 0.85-1.56 | 8.66% | -11.12% | 24.92% |
| **Overall** | 960,448 (34.65) | 1,811,276 (65.35) | 1.33 | 1.32-1.33 | 16.89% | 16.69% | 17.09% |
| **South** | | | | | | | |
| **1** | 64,066 (52.37) | 58,266 (47.63) | 1.21 | 1.19-1.23 | 12.07% | 11.12% | 13.00% |
| **2** | 49,640 (21.98) | 176,223 (78.02) | 1.61 | 1.58- 1.63 | 26.85% | 26.15% | 27.55% |
| **3** | 97,914 (81.23) | 22,623 (18.77) | 1.49 | 1.46-1.53 | 22.97% | 21.71% | 24.20% |
| **4** | 55,353 (44.58) | 68,804 (55.42) | 1.72 | 1.70-1.75 | 29.91% | 29.15% | 30.67% |
| **5** | 29,605 (10.55) | 251,067 (89.45) | 1.57 | 1.54-1.60 | 25.29 | 24.37 | 26.21 |
| **6** | 892 (4.63) | 18,381 (95.37) | 1.20 | 1.06- 1.36 | 9.74% | 3.23% | 15.81% |
| **7** | 1,779 (7.94) | 20,627 (92.06) | 1.24 | 1.14-1.35 | 11.70% | 7.27% | 15.92% |
| **8** | 1,860 (8.99) | 18,830 (91.01) | 1.97 | 1.72-2.26 | 13.69% | 11.11% | 16.19% |
| **9** | 62 (2.50) | 2,417 (97.50) | 1.38 | 0.88- 2.16 | 18.42% | -8.93% | 38.90% |
| **Overall** | 301,171 (32.09) | 637,238 (67.91) | 1.49 | 1.48-1.50 | 22.88% | 22.56% | 23.20% |
| **Central-West** | | | | | | | |
| **1** | 41,807 (42.93) | 55,568 (57.07) | 1.12 | 1.10-1.138 | 7.12% | 5.94% | 8.30% |
| **2** | 16,297 (19.88) | 65,685 (80.12) | 1.67 | 1.63-1.712 | 28.30% | 27.06% | 29.52% |
| **3** | 71,918 (73.60) | 25,798 (26.40) | 1.42 | 1.38- 1.449 | 20.30% | 19.07% | 21.51% |
| **4** | 19,900 (39.20) | 30,859 (60.80) | 1.76 | 1.71-1.805 | 30.77% | 29.52% | 32.01% |
| **5** | 16,757 (9.38) | 161,834 (90.62) | 1.57 | 1.54-1.60 | 25.29 | 24.37 | 26.21 |
| **6** | 646 (9.09) | 6,457 (90.91) | 1.06 | 0.92-1.23 | 3.40% | -5.27% | 11.35% |
| **7** | 1,281 (11.55) | 9,814 (88.45) | 1.26 | 1.14-1.38 | 12.94% | 7.61% | 17.96% |
| **8** | 825 (7.78) | 9,783 (92.22) | 1.77 | 1.47-2.12 | 15.44% | 10.77% | 19.86% |
| **9** | 18 (1.87) | 945 (98.13) | 1.43 | 0.67-3.04 | 19.05% | -26.32% | 48.12% |
| **Overall** | 169,449 (31.60) | 366,743 (68.40) | 1.34 | 1.33-1.35 | 17.49% | 17.01% | 17.97% |
| CS: Cesarean section; PAF: population attributable fraction; The early-term birth (37 and 38 weeks gestation) was compared with births at 39 to 41 weeks gestation; OR (odds ratio) from a logistic regression in which vaginal deliveries are the comparison group; *adjusted by PSM covariates; ** 0·1 width caliper; Robson groups: 1 (Nulliparous women with a single cephalic pregnancy, ≥ 37 weeks gestation in spontaneous labor); 2 (Nulliparous women with single cephalic pregnancy, ≥ 37 weeks gestation who either had labor induced or were delivered by cesarean section before labor); 3 (Multiparous women without a previous uterine scar, with a single cephalic pregnancy, > 37 weeks gestation, in spontaneous labor); 4 (Multiparous women without a previous uterine scar, with single cephalic pregnancy, ≥ 37 weeks gestation who either had labor induced or were delivered by cesarean section before labor); 5 (All multiparous women with at least one previous CS, with a single cephalic pregnancy, ≥ 37 weeks gestation); 6 (All nulliparous women with a single breech pregnancy); 7 (All multiparous women with a single breech pregnancy, including women with a previous CS); 8 (All women with multiple pregnancies, including women with a previous CS); 9 (All women with a single pregnancy with a transverse or oblique lie, including women with previous CS(s). | | | | | | | |

| **Table S6.** Sensitivity analyses for early-term births by type of delivery according to the Robson classification in Brazil, 2012-2019 (n=17,081,685) | | | | | | |
| --- | --- | --- | --- | --- | --- | --- |
| **Robson Groups** | **Unadjusted analysis** | | **Adjusted analysis*** | | **PSM**** | |
|  | **OR** | **CI 95%** | **OR** | **CI 95%** | **OR** | **CI 95%** |
| **1** | 1.14 | 1.13-1.14 | 1.06 | 1.06-1.07 | 1.05 | 1.04-1.05 |
| **2** | 1.58 | 1.58-1.59 | 1.37 | 1.37-1.38 | 1.39 | 1.38-1.40 |
| **3** | 1.31 | 1.30-1.32 | 1.29 | 1.28-1.30 | 1.31 | 1.30-1.32 |
| **4** | 1.72 | 1.71-1.72 | 1.58 | 1.57-1.59 | 1.58 | 1.56-1.59 |
| **5** | 1.62 | 1.61-1.63 | 1.40 | 1.39-1.41 | 1.34 | 1.33-1.35 |
| **6** | 1.25 | 1.21-1.30 | 1.14 | 1.10-1.18 | 1.14 | 1.09-1.19 |
| **7** | 1.33 | 1.30-1.36 | 1.19 | 1.17-1.21 | 1.18 | 1.15-1.22 |
| **8** | 2.03 | 1.97-2.09 | 1.50 | 1.46-1.55 | 1.44 | 1.38-1.50 |
| **9** | 1.19 | 1.03-1.36 | 1.13 | 0.98-1.31 | 1.18 | 1.15-1.22 |
|  |  |  |  |  |  |  |
| **All groups** | 1.56 | 1.56-1.56 | 1.39 | 1.39-1.40 | 1.33 | 1.33-1.34 |
| The early-term birth (37 and 38 weeks gestation) was compared with births at 39 to 41 weeks gestation. OR (odds ratio) from a logistic regression in which vaginal deliveries are the comparison group; *adjusted by PSM (propensity score matching) covariates: race/ethnicity, maternal education, marital status, number of prenatal appointments, maternal age at delivery, newborns` sex and year of birth; ** 0·05 width caliper (0·1 in the primary analyses); Robson groups: 1 (Nulliparous women with a single cephalic pregnancy, ≥ 37 weeks gestation in spontaneous labor); 2 (Nulliparous women with single cephalic pregnancy, ≥ 37 weeks gestation who either had labor induced or were delivered by cesarean section before labor); 3 (Multiparous women without a previous uterine scar, with a single cephalic pregnancy, > 37 weeks gestation, in spontaneous labor); 4 (Multiparous women without a previous uterine scar, with single cephalic pregnancy, ≥ 37 weeks gestation who either had labor induced or were delivered by cesarean section before labor); 5 (All multiparous women with at least one previous CS, with a single cephalic pregnancy, ≥ 37 weeks gestation); 6 (All nulliparous women with a single breech pregnancy); 7 (All multiparous women with a single breech pregnancy, including women with a previous CS); 8 (All women with multiple pregnancies, including women with a previous CS); 9 (All women with a single pregnancy with a transverse or oblique lie, including women with previous CS(s). | | | | | | |
